# Psychometric Properties of the Bangla Version of the Stress and Anxiety to Viral Epidemics-6 Items Scale Among the General Population in Bangladesh

**DOI:** 10.3389/fpsyt.2022.804162

**Published:** 2022-02-17

**Authors:** Oli Ahmed, Kazi Nur Hossain, Fatema Akhter Hiramoni, Rumana Ferdousi Siddique, Seockhoon Chung

**Affiliations:** ^1^Department of Psychology, University of Chittagong, Chattogram, Bangladesh; ^2^Research School of Population Health, Australian National University, Canberra, ACT, Australia; ^3^Department of Psychology, Jagganath University, Dhaka, Bangladesh; ^4^Department of Economics, Sheikh Hasina University, Netrokona, Bangladesh; ^5^Department of Psychology, University of Dhaka, Dhaka, Bangladesh; ^6^Department of Psychiatry, Asan Medical Center, University of Ulsan College of Medicine, Seoul, South Korea

**Keywords:** COVID-19, epidemics, anxiety, stress, psychological

## Abstract

**Background:**

Any disease outbreak creates psychological stress and anxiety among the public [e.g., Coronavirus Disease 2019 (COVID-19)]. There are several scales that assess anxiety specifically related to the COVID-19 pandemic. The Stress and Anxiety to Viral Epidemics-6 items (SAVE-6) scale is a reliable and valid tool to assess anxiety in any viral pandemic. The present study aims to validate the SAVE-6 scale in the Bangla language and culture, to assess such anxiety among the general Bangladeshi people.

**Methods:**

The SAVE-6 scale was translated into Bangla from English using the forward-backward translation procedure. A total of 357 Bangladeshi citizens participated via an online structured questionnaire. The items included questions on personal information, COVID-19 and vaccination, psychiatric history, the Bangla version of the SAVE-6 scale, the Generalized Anxiety Disorder-7 items (GAD-7) scale, and the Patient Health Questionnaire-9 items (PHQ-9) scale.

**Results:**

Both exploratory factor analysis and confirmatory factor analysis (CFA) were used to explore and confirm the single factor structure of the SAVE-6 scale in Bangla to be the same as that of the SAVE-6 scale. Multigroup CFA revealed invariance across sex, experience of being quarantined, experience of being infected, and presence of depression. Item analysis results showed good discrimination indices and internal consistency and reliability. The graded response model outputs also confirmed the validity and reliability of this scale, which had significant correlations with the GAD-7 and PHQ-9.

**Conclusion:**

Overall, the Bangla version of the SAVE-6 is a psychometrically good scale to assess viral pandemic-related anxiety.

## Introduction

Since the first report of the coronavirus Disease 2019 (COVID-19) case in December 2019 in Wuhan, Hubei Province, China^1^, the virus spread rapidly worldwide. During 2 years of COVID-19, 281,808,270 confirmed cases and 5,411,759 deaths had been reported worldwide^1^. In Bangladesh, the first COVID-19 case was identified on March 08, 2020; 10 days later, the first related death was reported^1^. In the initial emergency response to control the community transmission of COVID-19, the Bangladesh government shut down all educational institutions on March 17, 2020, and imposed a countrywide lockdown on March 26, 2020, which was extended to May 31. At the beginning of 2021, Bangladesh experienced a second wave of the pandemic caused by the Delta variant of the virus. The Bangladesh government imposed a nationwide lockdown once again from April 05 to July 14, 2021. After a short pause, the government re-imposed the nationwide lockdown from July 23 to August 10, 2021^2^. By October 14, 2021, 1,564,485 people in Bangladesh had been affected by COVID-19 and 27,737 had died from the disease^3^.

The current COVID-19 pandemic has affected the psychological and physical health of people worldwide. Many countries have imposed lockdown measures as a first line of defense to contain the spread of the virus. During these lockdown measures, lives became stagnant and psychological distress increased (1, 2), while sleep schedules were altered (3). Numerous studies have reported a higher prevalence of COVID-19-related stress, anxiety, depression, poor sleep quality, and sleep problems (4–9). Ahmed et al. (10) reported that approximately one-third of the people were highly anxious about COVID-19 and had higher anxiety and depression symptoms compared to pre-pandemic levels. In a longitudinal study, Ramiz et al. (11) reported increased anxiety symptoms and decreased mental wellbeing among French people. Women, younger individuals, and the older adults are more vulnerable in this pandemic. In another comprehensive longitudinal study, Wu et al. (12) reported increased anxiety and depressive symptoms among Chinese people.

Several rating scales for measuring the anxiety response to COVID-19 have been developed (13–19). The Stress and Anxiety to Viral Epidemics-6 items (SAVE-6) scale is a self-rating scale that can measure one's anxiety response specifically to the viral epidemic (20). It was derived from the SAVE-9 scale, which was developed to measure work-related stress and anxiety responses of healthcare workers to the viral epidemic (21). The SAVE-6 scale was validated among the general population in Korea (20), Lebanon (22), and the United States (23), and among special populations such as medical students (24), public workers (25), or cancer patients (26). In this study, we attempt to explore the validity and reliability of the Bangla version of the SAVE-6 scale.

## Methods

### Measures

#### Sociodemographic Characteristics

This study included the Bangladeshi general population as the study population. Participants were recruited using a snowball sampling technique. The data of this study were collected through an online survey between September 16, 2021, and October 04, 2021. The online survey link was distributed via social media (e.g., Messenger and WhatsApp) and e-mail. We emailed individuals with whom we had frequent email contact. In addition, we emailed through a university group email by which we were able to reach a large number of recipients. Meanwhile, we distributed the survey link to our Messenger and WhatsApp contacts, as well as sharing this link on Facebook and inviting our Facebook friends to participate in this survey. A total of 399 adult Bangladeshi individuals enrolled in this study. Among them, four disagreed to participate. After excluding missing observations for age, sex, and the scales utilized, data from 357 participants were used in this study. Participation was voluntary and no compensation was provided for participation. This study was approved by the Institutional Review Board of the University of Ulsan (2001R0043), and written informed consent for participation was waived.

#### Stress and Anxiety to Viral Epidemics-6 Items

The SAVE-6 scale consists of six items, each rated on a 5-point scale, from 0 (never) to 4 (always). The total score may range from 0 to 24, with higher scores reflecting higher levels of anxiety response to the viral epidemic. The SAVE-6 scale was derived from the original SAVE-9 scale developed for measuring work-related stress and anxiety responses of healthcare workers to the viral epidemic (21). In this study, we adopted the translation and back-translation process to develop the Bangla version of the SAVE-6 scale. First, two bilingual experts translated the questionnaire into the Bangla language from English. The two translated Bangla versions were then synthesized into one. Next, the synthesized version was back translated into English by two other bilingual experts. These two back translations were again synthesized into one and compared with the original English version to identify any discrepancy in meaning. As there was no discrepancy in meaning, the translated Bangla version was continued to the final study.

#### Patient Health Questionnaire-9 Items

The PHQ-9 scale is a self-rating scale for measuring the severity of depression (27). Each of the 9 items can be rated from 0 (not at all) to 3 (nearly every day). The total score ranges from 0 to 27, with higher scores reflecting more severe levels of depression. The cut-offs for depression are 0–4 (minimal), 5–9 (mild), 10–14 (moderate), 15–19 (moderately severe), and 20–27 (severe). In this study, we applied the Bangla version of the PHQ-9 (28), and the Cronbach's alpha was 0.889 in this sample.

#### Generalized Anxiety Disorder-7 Items

The GAD-7 scale is a self-rating scale for measuring the severity of general anxiety (29). Each of the seven items can be rated from 0 (not at all) to 3 (nearly every day). The total score ranges from 0 to 21, and a higher score reflects more severe levels of general anxiety. The cut-offs for anxiety are 0–4 (minimal), 5–9 (mild anxiety), 10–14 (moderate), and 15–21 (severe). In this study, we applied the Bangla version of the GAD-7 (30), and acquired a Cronbach's alpha of 0.921 in this sample.

### Statistical Analysis

We conducted several statistical tests under classical and modern test theory approaches to assess the psychometric properties of the Bangla version of the SAVE-6 scale. Skewness (acceptable range = ±2) and kurtosis (acceptable range = ±7) (31) were utilized to assess the normality assumption. Next, exploratory factor analysis (EFA) and confirmatory factor analysis (CFA) were used to explore the number of latent factors of the SAVE-6 scale. In the EFA, data adequacy was assessed through the Kaiser–Meyer–Olkin (KMO) value, whereas sampling adequacy was assessed through Bartlett's test of sphericity. A scree plot and EFA, using the maximum likelihood method with a Pearson's correlation matrix in oblimin rotation, were conducted to explore the factors of the Bangla version of the SAVE-6. In addition, CFA with a diagonally weighted least squares (DWLS) estimator was used to examine the factor structure of the Bangla version of SAVE-6. Model fit was assessed through the χ^2^/df ratio based on the original chi-square, comparative fit index (CFI), Tucker–Lewis index (TLI), root-mean-square-error of approximation (RMSEA), and standardized root-mean-square residual (SRMR) values. A series of multi-group CFA with configural invariance testing was run to determine whether the Bangla SAVE-6 scale assessed the anxiety response across sex, experience of being quarantined, experience of being infected, and presence of depression, and confirmed whether the CFA with metric and scale constraints are sufficiently corroborated by the model fit. Then, modern test theory assumptions (i.e., unidimensionality, local dependence, and monotonicity) were estimated. Unidimensionality, local dependence, and monotonicity were assessed using Loevinger's H coefficient, *p*-values (adjusted for false discovery rate) of G^2^, and the number of significant violations and *Crit* value, respectively. The graded response model (GRM), a modern test theory model for polytomous items, was run to assess the psychometric properties of the scale. Under the GRM, item fits were first assessed through S-χ^2^ and its *p*-values (adjusted for false discovery rate). Next, the slope parameters (α) and threshold parameters (b) of the items assessed, as well as scale information curve of the SAVE-6 scale, were extracted. Loevinger's H coefficient and monotonicity were estimated through the R package Mokken version 3.0.6. Local dependence and GRM were run through the R package mirt version 1.34.

Item analysis was conducted to estimate internal consistency reliability [Cronbach's alpha, McDonald's omega, and split-half reliability (odd-even)]. Moreover, the floor and ceiling effect, mean inter-item correlation, corrected item-total correlation, standard error of measurement, Ferguson's delta, item response theory (IRT) reliability, and correlation coefficient were calculated. The standard error of measurement (SEM) was computed using the formula: SEM = standard deviation ^*^ [SQRT (1 – reliability)]. Pearson product-moment correlation was run to estimate the correlation between the SAVE-6, and GAD-7 and PHQ-9 scales. The two-independent samples *t*-test was run to assess the mean differences in the SAVE-6 scores between those having depression (PHQ-9 ≥ 10) and those without (PHQ-9 <10), and between those having anxiety (GAD-7 ≥ 10) and no anxiety (GAD-7 <10). Microsoft Excel 365, IBM Statistical Package for the Social Sciences version 26, and Rstudio were utilized for statistical analysis.

## Results

### Demographic Characteristics

Among the 357 participants, 185 (51.8%) were male (Table 1), 44.8% were single, and 71.4% were living in the city. The participants' mean age was 37.03 ± 15.99 years, 34.2% experienced being quarantined, 19.3% were infected, and 57.7% got vaccinated. Moreover, 34.7% of the participants had psychiatric history, and those in need of help for their psychiatric symptoms were 39.8%.

**Table 1:** Clinical characteristics of the participants (*N* = 357).

**Variables**	**Mean ±SD, *N* (%)**
**Sex (male)**	185 (51.8%)
**Age (in years)**	37.03 ± 15.99
**Marital status**	
Single	160 (44.8%)
Married, without children	34 (9.5%)
Married, with children	159 (44.5%)
**Living area**	
City	255 (71.4%)
Village	102 (28.6%)
**Questions on COVID-19**	
Did you experience being quarantined due to infection with COVID-19? (Yes)	122 (34.2%)
Did you experience being infected with COVID-19? (Yes)	69 (19.3%)
Did you get vaccinated? (Yes)	206 (57.7%)
(Among participants who did not get vaccinated. *N* = 151)	138 (91.4%)
Do you want to get vaccinated if it is available? (Yes)	
**Psychiatric history**	
Have you experienced or been treated for depression, anxiety, or insomnia? (Yes)	124 (34.7%)
Now, do you think you are depressed or anxious, or do you need help for your mood state? (Yes)	142 (39.8%)
**Rating scales**	
Patient health questionnaire-9 items	8.7 ± 6.3
Generalized anxiety disorders-7 items	6.9 ± 5.9
Stress and anxiety to viral epidemics-6 items	9.6 ± 5.2

### Initial Exploratory Factor Analysis

The normality assumption for all 6 items of the SAVE-6 scale was checked through the skewness and kurtosis within the range of ±2 (Table 2). The data were observed to be suitable for factor analysis based on a KMO measure of 0.85 and Bartlett's test of sphericity value of *p* < 0.001. A scree plot and EFA showed that the single factor model of the Bangla version of the SAVE-6 scale was a good fit [χ^2^ = 34.517, df = 9, *p* < 0.001, TLI = 0.943, RMSEA = 0.090 (0.059, 0.122)].

**Table 2:** Item properties of the Bangla version of the SAVE-6 scale.

**Items**	**Response scale**					**Descriptive**				**CITC**	**CID**	**Factor loading (95% CI)**
	**0**	**1**	**2**	**3**	**4**	***M***	***SD***	**Skewness**	**Kurtosis**			
Item 1	21.0%	26.3%	36.4%	13.4%	2.8%	1.51	1.05	0.15	−0.65	0.63	0.80	0.70 (0.62, 0.79)
Item 2	28.9%	26.3%	30.0%	10.9%	3.9%	1.35	1.12	0.42	−0.61	0.66	0.79	0.76 (0.67, 0.85)
Item 3	17.4%	26.1%	28.3%	19.6%	8.7%	1.76	1.20	0.17	−0.88	0.74	0.77	0.85 (0.75, 0.94)
Item 4	19.0%	27.2%	25.8%	18.2%	9.8%	1.73	1.24	0.24	−0.92	0.59	0.80	0.65 (0.56, 0.73)
Item 5	49.0%	19.9%	16.2%	9.8%	5.0%	1.02	1.23	0.95	−0.24	0.48	0.83	0.52 (0.44, 0.60)
Item 6	11.5%	18.8%	24.9%	24.9%	19.9%	2.23	1.28	−0.20	−1.02	0.53	0.82	0.57 (0.50, 0.65)

*0 = never, 1 = rarely, 2 = sometimes, 4 = often, 5 = always; M, mean; SD, standard deviation; CITC, corrected item-total correlation; CID, Cronbach's alpha if item deleted; CI, confidence interval*.

### Confirmatory Factor Analysis

**Figure 1:**
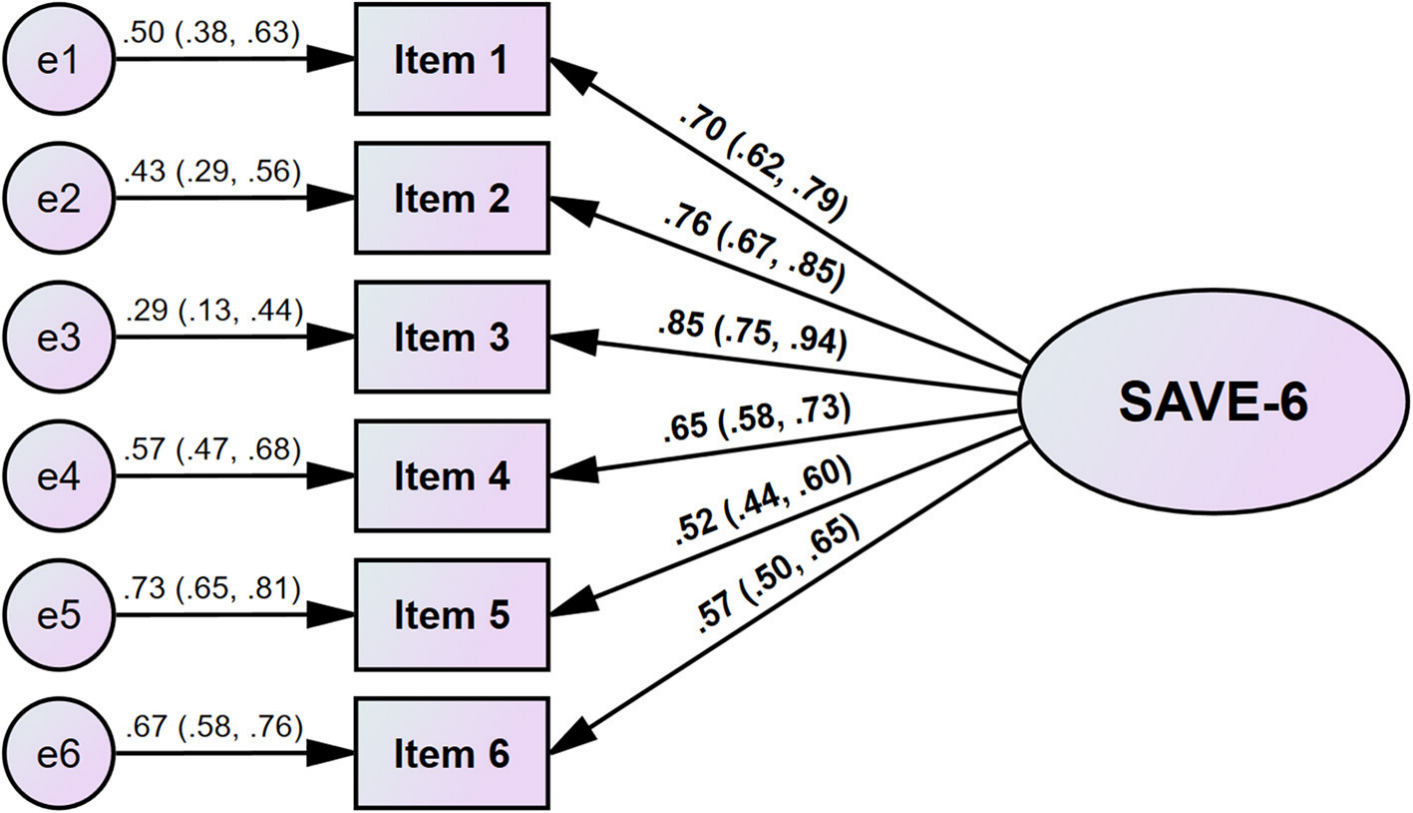
Factor structure of the Bangla version of the SAVE-6 scale.

The CFA of the SAVE-6 scale showed good model fit (χ^2^/df = 1.081, CFI = 0.999, TLI = 0.999, RMSEA = 0.015, and SRMR = 0.037). Factor loadings ranged between 0.52 (0.44, 0.60) and 0.85 (0.75, 0.94) (Table 2; Figure 1). The multi-group CFA revealed that the SAVE-6 scale could measure anxiety response in the same way across sex (CFI = 1.000, TLI = 1.000, RMSEA = 0.000, RSMR = 0.045), experience of being quarantined (CFI = 1.000, TLI = 1.000, RMSEA = 0.000, RSMR = 0.048), experience of being infected (CFI = 1.000, TLI = 0.999, RMSEA = 0.013, RSMR = 0.069), or presence of depression (CFI = 1.000, TLI = 1.000, RMSEA = 0.000, RSMR = 0.051). Multigroup CFA with metric or scale invariant model also showed similar results.

### Graded Response Model Analysis

Information about IRT assumptions are presented in Table 3 and Supplementary Table 1. Loevinger's H coefficient (0.49; Table 3) suggests that the Bangla version of the SAVE-6 scale is moderately unidimensional. Non-significant *p*-values of G^2^ (Supplementary Table 2) suggest the absence of local dependence between items of the Bangla version of the SAVE-6 scale. The absence of significant violation and the low value of the *Crit* statistic for all items suggest that the monotonicity assumption is tenable. These results suggest that all the IRT model assumptions were met. Supplementary Table 1 presents the item fit statistics of the Bangla version of the SAVE-6 scale. After controlling the false discovery rate (FDR), the *p*-values of the S-χ^2^ suggest that all items fit well in the scale, indicating that all the items belong to the Bangla version of the SAVE-6 scale. The slope/discrimination parameters (α) range from 1.251 to 3.182 (mean = 2.025) (Supplementary Table 1). The slopes of items 5 and 6 were moderate, that of item 4 was high, and those of the rest were very high. All the items provide reasonable information and are efficient in discriminating among individuals in anxiety and stress, as assessed by the Bangla version of the SAVE-6 scale. Among the items, item 3 provides the most information whereas item 5 provides the least. The threshold coefficients (b) in Supplementary Table 2 suggest that a higher latent trait or theta is required to endorse items 2 and 5 compared to the other items. Regarding items 2 and 5, only b_1_ coefficients are negative and the rest are positive, suggesting that an above average level of latent trait or theta is required to endorse Likert-type response options: from “sometimes” to “often.” The scale information curve (Supplementary Figure 1) provides an understanding about information provided by the Bangla version of the SAVE-6 scale. From this curve, this scale provides more information about people between the −0.25 and 1.75 θ level. There are two peaks in the curve, which may be due to the polytomous nature of the data.

**Table 3:** Scale-level psychometric properties of the Bangla version of the SAVE-6 scale.

**Psychometric properties**	**Scores**	**Suggested cut off**
Floor effect	3.6%	15%
Ceiling effect	0%	15%
Mean inter-item correlation	0.45	Between 0.15 and 0.50
Cronbach's alpha	0.83	≥0.7
McDonald's Omega	0.84	≥0.7
Split-half reliability (odd-even)	0.86	≥0.7
Standard error of measurement	2.16	Smaller than *SD* (5.25)/2
Ferguson delta	0.99	≥0.9
Loevinger's *H* coefficients	0.49	–
*Rho* coefficient	0.83	≥0.7
IRT reliability	0.87	≥0.7
**Model fits of confirmatory factor analysis**		
*χ^2^* (df, *p*-value), χ^2^/df	9.725 (9, 0.373), 1.081	Non-significant, <3
CFI	0.999	>0.95
TLI	0.999	>0.95
RMSEA (90% CI value) (*p*-value)	0.015 (0.000, 0.063) (0.857)	<0.08
SRMR	0.037	<0.08

*IRT, item response theory; CFI, comparative fit index; TLI, Tucker–Lewis index; RMSEA, root-mean-square-error of approximation; SRMR, standardized root-mean-square residual*.

### Reliability and Evidence Based on Relations to Other Variables

The Bangla version of the SAVE-6 scale showed good reliability for internal consistency [Cronbach's alpha = 0.84; McDonald's Omega = 0.84; split-half reliability (odd-even) = 0.86]. If an item is dropped, Cronbach's alpha was measured as 0.77–0.83. The mean inter-item correlation (0.45) was between the recommended range (0.15–0.50). This scale also had good IRT reliability (0.87) and rho coefficient (0.83), as well as good discriminatory power (Ferguson's delta = 0.99). The total score the SAVE-6 scale was significantly correlated with those of the PHQ-9 [*r* = 0.332 (95% CI, 0.238, 0.419), *p* < 0.001] and GAD-7 [*r* = 0.376 (95% CI, 0.285, 0.460), *p* < 0.001] scores. The Bangla version of the SAVE-6 score was significantly higher among participants with depression [PHQ-9 ≥ 10, *t*_(373)_ = 6.762, *p* < 0.001] and anxiety [GAD-7 ≥ 10, *t*_(368)_ = 7.483, *p* < 0.001].

## Discussion

This study aimed to assess the psychometric properties of the Bangla version of the SAVE-6 scale among the general Bangladeshi population to assess its efficacy in measuring anxiety in a viral pandemic such as COVID-19. Online data were collected from the general population in Bangladeshi. Both the EFA and CFA results explored and confirmed the single factor structure of the scale. This finding is consistent with those of other studies that assessed this scale's factor structure (20, 22–25). Items 5 and 6 had slightly lower, albeit acceptable, factor loadings. The survey data were collected when Bangladesh returned to relative normalcy after the end of the second wave of COVID-19, which is a possible reason for these lower factor loadings. The SAVE-6 scale revealed the same latent construct between men and women, those who experienced being quarantined and otherwise, those who were and were not infected, and those with and without depressive symptoms. The results are also consistent with other studies conducted to assess the psychometric properties of the SAVE-6 scale across different cultures and groups (23–25).

In this study, we also assessed the efficiency of the Bangla version of the SAVE-6 scale through the modern test theory model GRM. Similar to the EFA and CFA results, the unidimensionality test (Loevinger's H coefficient) confirmed the single factor structure of the scale. The item fit values confirmed that all the items belonged to the SAVE-6 scale. The slope parameter values suggested that all the items were efficient to discriminate between high and low scores in the Bangla version of the SAVE-6 scale. Similar to the factor loadings in the CFA, items 5 and 6 had moderate slope parameters. This might be due to the same reason as that of the low factor loadings of items 5 and 6. The threshold parameters showed items 2 and 5 to be more difficult compared to the other four. This scale provides more information about people having latent traits around the “0” theta level.

The item analysis results suggest good internal consistency reliability, which is consistent with previous studies (20, 23–25). Furthermore, the scale items also had good discrimination indices (corrected item-total correlations). These items can discriminate between low and high scorers in the Bangla version of the SAVE-6 scale. The Bangla version of the SAVE-6 scale had moderately positive correlations with anxiety and depression symptoms. Similar results have been reported in previous studies (22, 24, 25). Overall, the Bangla SAVE-6 scale is a psychometrically good scale to assess anxiety related to the viral pandemic among the general population in Bangladesh.

This study has several limitations. First, we collected the study data via an online questionnaire; consequently, only educated people with Internet-enabled and digital devices could participate in the survey. Potential users of this scale should consider this issue. Second, the data of this study were collected at the end of the second wave of the COVID-19 pandemic in Bangladesh. The general Bangladeshi population are reluctant to conform to the World Health Organization recommendations due to prolonged lockdowns. Therefore, the timing of the viral pandemic might influence the results. Third, the data in this study were self-rated, and might be subject to certain biases, such as social desirability bias. Fourth, a screening norm was not established in this study. Although the SAVE-6 scale in other cultures and languages established a cut-off score, we did not do so in this study.

Overall, the Bangla version of the SAVE-6 scale is a reliable and valid scale to assess anxiety among the general Bangladeshi population. Both classical and modern test theory approaches support the appropriateness of this scale to assess anxiety during a viral pandemic among the Bangladeshi people. This scale is beneficial to researchers and mental health practitioners. Researchers could use this scale to assess viral pandemic anxiety and its relation to psycho-social factors, while mental health practitioners can formulate interventions to cope with viral pandemic anxiety using this scale.

## Data Availability Statement

The raw data supporting the conclusions of this article will be made available by the authors, without undue reservation.

## Ethics Statement

This study was approved by the Institutional Review Board of the University of Ulsan (2001R0043) and the obtaining the written informed consent for participation was waived. Written informed consent for participation was not required for this study in accordance with the national legislation and the institutional requirements.

## Author Contributions

OA and SC: conceptualization, formal analysis, methodology, and writing—original draft. OA, KH, FH, and RS: data curation. SC, FH, and RS: investigations. KH: project administration. OA: visualization. All authors writing—review and editing. All authors contributed to the article and approved the submitted version.

## Conflict of Interest

The authors declare that the research was conducted in the absence of any commercial or financial relationships that could be construed as a potential conflict of interest.

## Publisher's Note

All claims expressed in this article are solely those of the authors and do not necessarily represent those of their affiliated organizations, or those of the publisher, the editors and the reviewers. Any product that may be evaluated in this article, or claim that may be made by its manufacturer, is not guaranteed or endorsed by the publisher.

## Footnotes

^1^Available online at: https://covid19.who.int/ (accessed January 4, 2022).

^2^Available online at: https://betterwork.org/portfolio/covid-timeline-in-bangladesh/ (accessed October 15, 2021).

^3^Available online at: https://covid19.who.int/region/searo/country/bd (accessed October 14, 2021).

## References

[1] Brooks SK Webster RK Smith LE Woodland L Wessely S Greenberg N. The psychological impact of quarantine and how to reduce it: rapid review of the evidence. Lancet. (2020) 395:912–20. 10.1016/S0140-6736(20)30460-8 32112714 PMC7158942

[2] Burke T Berry A Taylor LK Stafford O Murphy E Shevlin M. Increased psychological distress during COVID-19 and quarantine in Ireland: a national survey. J Clin Med. (2020) 9:3481. 10.3390/jcm9113481 33126707 PMC7693396

[3] Cellini N Canale N Mioni G Costa S. Changes in sleep pattern, sense of time and digital media use during COVID-19 lockdown in Italy. J Sleep Res. (2020) 29:e13074. 10.1111/jsr.13074 32410272 PMC7235482

[4] Ahmed MZ Ahmed O Aibao Z Hanbin S Siyu L Ahmad A. Epidemic of COVID-19 in China and associated psychological problems. Asian J Psychiatr. (2020) 51:102092. 10.1016/j.ajp.2020.102092 32315963 PMC7194662

[5] Beck F Leger D Fressard L Peretti-Watel P Verger P Coconel G. Covid-19 health crisis and lockdown associated with high level of sleep complaints and hypnotic uptake at the population level. J Sleep Res. (2021) 30:e13119. 10.1111/jsr.13119 32596936 PMC7361195

[6] Jahrami H BaHammam AS Bragazzi NL Saif Z Faris M Vitiello MV. Sleep problems during the COVID-19 pandemic by population: a systematic review and meta-analysis. J Clin Sleep Med. (2021) 17:299–313. 10.5664/jcsm.8930 33108269 PMC7853219

[7] Li Y Qin Q Sun Q Sanford LD Vgontzas AN Tang X. Insomnia and psychological reactions during the COVID-19 outbreak in China. J Clin Sleep Med. (2020) 16:1417–8. 10.5664/jcsm.8524 32351206 PMC7446072

[8] Marelli S Castelnuovo A Somma A Castronovo V Mombelli S Bottoni D. Impact of COVID-19 lockdown on sleep quality in university students and administration staff. J Neurol. (2021) 268:8–15. 10.1007/s00415-020-10056-6 32654065 PMC7353829

[9] Wang C Pan R Wan X Tan Y Xu L Ho CS. Immediate psychological responses and associated factors during the initial stage of the 2019 coronavirus disease (COVID-19) epidemic among the general population in China. Int J Environ Res Public Health. (2020) 17:1729. 10.3390/ijerph17051729 32155789 PMC7084952

[10] Ahmed O Ahmed MZ Alim S Khan M Jobe MC. COVID-19 outbreak in Bangladesh and associated psychological problems: an online survey. Death Stud. (2020). 10.1080/07481187.2020.1818884. [Epub ahead of print]. 32915701

[11] Ramiz L Contrand B Rojas Castro MY Dupuy M Lu L Sztal-Kutas C. A longitudinal study of mental health before and during COVID-19 lockdown in the French population. Global Health. (2021) 17:29. 10.1186/s12992-021-00682-8 33752717 PMC7982911

[12] Wu S Zhang K Parks-Stamm EJ Hu Z Ji Y Cui X. Increases in anxiety and depression during COVID-19: a large longitudinal study from China. Front Psychol. (2021) 12:706601. 10.3389/fpsyg.2021.706601 34295294 PMC8290070

[13] Ahorsu DK Lin CY Imani V Saffari M Griffiths MD Pakpour AH. The fear of COVID-19 scale: development and initial validation. Int J Ment Health Addict. (2020). 10.1007/s11469-020-00270-8. [Epub ahead of print]. PMC7100496 32226353

[14] Silva WAD de Sampaio Brito TR Pereira CR. COVID-19 anxiety scale (CAS): development and psychometric properties. Curr Psychol. (2020) 1–10. 10.1007/s12144-020-01195-0. [Epub ahead of print]. PMC7661558 33204058

[15] Lee SA. Coronavirus anxiety scale: a brief mental health screener for COVID-19 related anxiety. Death Stud. (2020) 44:393–401. 10.1080/07481187.2020.1748481 32299304

[16] Lee SA. How much “Thinking” about COVID-19 is clinically dysfunctional? Brain Behav Immun. (2020) 87:97. 10.1016/j.bbi.2020.04.067 32353520 PMC7185010

[17] Nikcevic AV Spada MM. The COVID-19 anxiety syndrome scale: development and psychometric properties. Psychiatry Res. (2020) 292:113322. 10.1016/j.psychres.2020.113322 32736267 PMC7375349

[18] Pakpour AH Griffiths MD Lin CY. Assessing psychological response to the COVID-19: the fear of COVID-19 scale and the COVID stress scales. Int J Ment Health Addict. (2021). 10.1007/s11469-020-00334-9. [Epub ahead of print]. PMC7259433 32837424

[19] Petzold MB Bendau A Plag J Pyrkosch L Maricic LM Rogoll J. Development of the COVID-19-anxiety questionnaire and first psychometric testing. BJPsych Open. (2020) 6:e91. 10.1192/bjo.2020.82 32812525 PMC7453355

[20] Chung S Ahn MH Lee S Kang S Suh S Shin WY. The stress and anxiety to viral epidemics-6 items (SAVE-6) scale: a new instrument for assessing the anxiety response of general population to the viral epidemic during the COVID-19 pandemic. Front Psychol. (2021) 12:669606. 10.3389/fpsyg.2021.669606 34149565 PMC8212887

[21] Chung S Kim H Ahn M Yeo S Lee J Kim K. Development of the stress and anxiety to viral epidemics-9 (SAVE-9) scale for assessing work-related stress and anxiety in healthcare workers in response to viral epidemics. J Korean Med Sci. (2021) 36:e319. 10.3346/jkms.2021.36.e319 34873885 PMC8648611

[22] Hong Y Yoo S Mreydem HW Abou Ali BT Sale NO Hammoudi SF. Factorial validity of the arabic version of the stress and anxiety to viral epidemics-6 items (SAVE-6) scale among the general population in Lebanon. J Korean Med Sci. (2021) 36:e168 10.3346/jkms.2021.36.e168 PMC8239423 34184434

[23] Lee S Lee J Yoo S Suh S Chung S Lee SA. The psychometric properties of the stress and anxiety to viral epidemics-6 items: a test in the U.S. general population. Front Psychiatry. (2021) 12:746244. 10.3389/fpsyt.2021.746244 34690844 PMC8526790

[24] Ahn J Lee J Hong Y Park J Chung S. Stress and anxiety to viral epidemics-6 for medical students: psychometric properties of the anxiety measure for the COVID-19 pandemic. Front Psychiatry. (2021) 12:705805. 10.3389/fpsyt.2021.705805 34413799 PMC8369005

[25] Park CHK Ju G Yi K Lee S Suh S Chung S. Application of stress and anxiety to viral epidemics-6 items (SAVE-6) to public workers for measuring their anxiety response during the COVID-19 pandemic. Front Psychiatry. (2021) 12:701543. 10.3389/fpsyt.2021.701543 34690826 PMC8528198

[26] Ahn MH Lee J Suh S Lee S Kim HJ Shin YW. Application of the stress and anxiety to viral epidemics-6 (SAVE-6) and coronavirus anxiety scale (CAS) to measure anxiety in cancer patient in response to COVID-19. Front Psychol. (2020) 11:604441. 10.3389/fpsyg.2020.604441 33329275 PMC7719621

[27] Kroenke K Spitzer RL Williams JB. The PHQ-9: validity of a brief depression severity measure. J Gen Intern Med. (2001) 16:606–13. 10.1046/j.1525-1497.2001.016009606.x 11556941 PMC1495268

[28] Naher R Reza-A-Rabby M Sharif F. Validation of patient health questionnaire-9 for assessing depression of adults in Bangladesh. Dhaka Univ J Biol Sci. (2021) 30:275–81. 10.3329/dujbs.v30i2.54652

[29] Spitzer RL Kroenke K Williams JB Lowe B. A brief measure for assessing generalized anxiety disorder: the GAD-7. Arch Intern Med. (2006) 166:1092–7. 10.1001/archinte.166.10.1092 16717171

[30] Faisal RA Jobe MC Ahmed O Sharker T. Mental health status, anxiety, and depression levels of Bangladeshi University students during the COVID-19 pandemic. Int J Ment Health Addict. (2021). 10.1007/s11469-020-00458-y. [Epub ahead of print]. PMC7781410 33424514

[31] Kim HY. Statistical notes for clinical researchers: assessing normal distribution (2) using skewness and kurtosis. Restor Dent Endod. (2013) 38:52–4. 10.5395/rde.2013.38.1.52 23495371 PMC3591587

