# Supplementary material for: Psychometric Properties of the Bangla Version of the Stress and Anxiety to Viral Epidemics-6 Items Scale Among the General Population in Bangladesh
Source: Front Psychiatry. 2022 Feb 17;13:804162. doi: 10.3389/fpsyt.2022.804162 (PMC8891513; doi:10.3389/fpsyt.2022.804162)
Supplement: Supplementary file 1 [file Data_Sheet_1.docx]

Supplementary Material

# Supplementary Table 1. Item fit, slope, and threshold parameters of the Bangla version of the SAVE-6 scale.

| Items | Item fits | | | Slope parameter (α) | Threshold parameter (b) | | | |
| --- | --- | --- | --- | --- | --- | --- | --- | --- |
|  | S-χ^2^ | Df | p value |  | b_1_ | b_2_ | b_3_ | b_4_ |
| Item 1 | 30.72 | 30 | .830 | 2.097 | -1.035 | -.059 | 1.289 | 2.473 |
| Item 2 | 17.86 | 27 | .908 | 2.698 | -.650 | .164 | 1.220 | 2.051 |
| Item 3 | 41.37 | 30 | .243 | 3.182 | -1.086 | -.153 | .697 | 1.530 |
| Item 4 | 35.98 | 39 | .830 | 1.618 | -1.273 | -.103 | .873 | 1.888 |
| Item 5 | 61.06 | 44 | .243 | 1.251 | -.051 | .783 | 1.702 | 2.787 |
| Item 6 | 35.06 | 40 | .830 | 1.305 | -1.981 | -.789 | .247 | 1.385 |
| Notes: p-values adjusted for false discovery rate (FDR) | | | | | | | | |

# Supplementary Table 2. Loevinger’s H coefficient, monotonicity, and G^2^ p values of items of the Bangla version of the SAVE-6 scale.

|  | ***H* coefficients** | **Monotonicity** | | | | **Local dependance G^2^ p values** | | | | |
| --- | --- | --- | --- | --- | --- | --- | --- | --- | --- | --- |
|  |  | **#ac** | **#vi** | **#zsig** | ***Crit*** | **Item1** | **Item2** | **Item3** | **Item4** | **Item5** |
| **Item1** | .71 | 22 | 0 | 0 | 0 |  |  |  |  |  |
| **Item2** | .71 | 16 | 0 | 0 | 0 | .181 |  |  |  |  |
| **Item3** | .66 | 21 | 0 | 0 | 0 | .181 | .181 |  |  |  |
| **Item4** | .59 | 21 | 0 | 0 | 0 | .181 | .181 | .181 |  |  |
| **Item5** | .53 | 19 | 0 | 0 | 0 | .181 | .181 | .181 | .181 |  |
| **Item6** | .49 | 24 | 0 | 0 | 0 | .181 | .181 | .181 | .181 | .183 |
| ac = active comparison, vi = violation, zsig = significant violation  Notes: p-values adjusted for false discovery rate (FDR) | | | | | | | | | | |


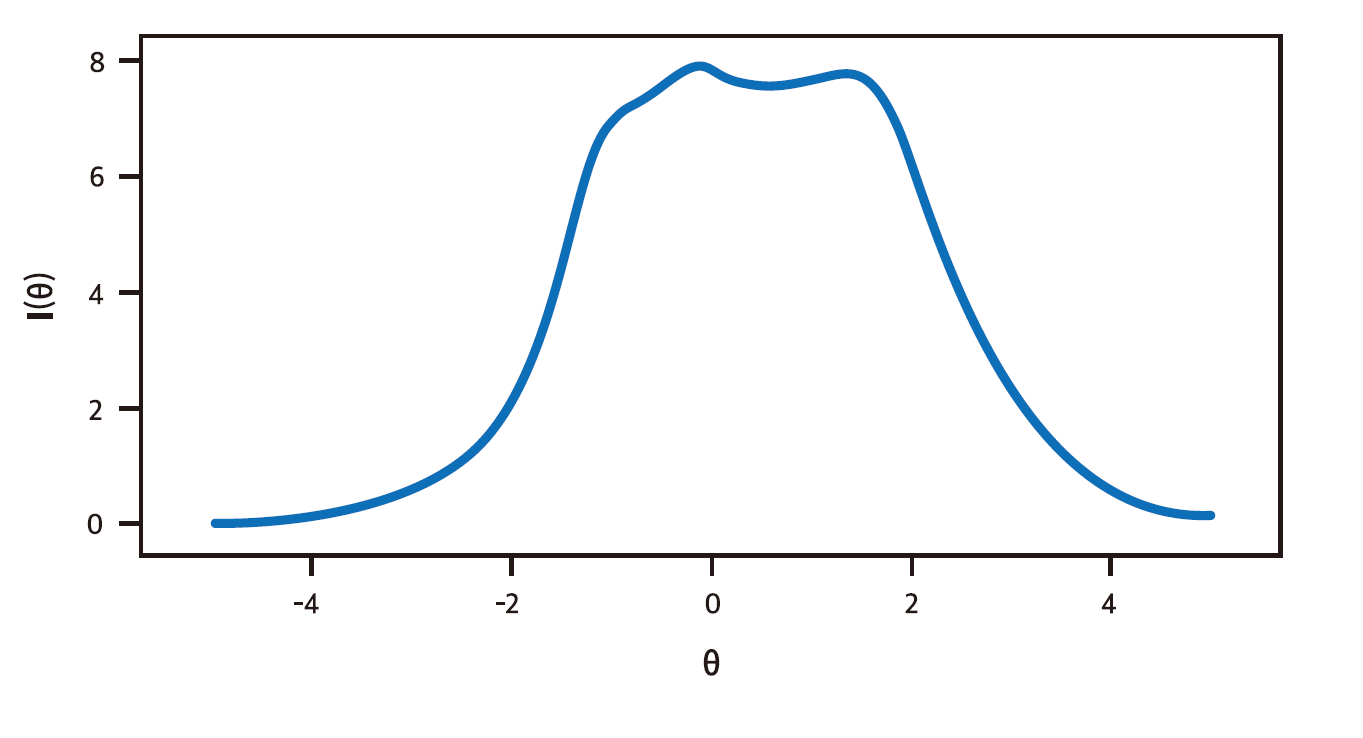


**Supplementary Figure 1.** Scale information curve of the SAVE-6.
